# Deletion of Trp53 and Rb1 in Ctsk‐expressing cells drives osteosarcoma progression by activating glucose metabolism and YAP signaling

**DOI:** 10.1002/mco2.131

**Published:** 2022-04-22

**Authors:** Yang Li, Shuting Yang, Yang Liu, Shuying Yang

**Affiliations:** ^1^ Department of Basic & Translational Sciences School of Dental Medicine University of Pennsylvania Philadelphia Pennsylvania USA; ^2^ College of Fisheries and Life Science Dalian Ocean University Dalian China; ^3^ Center for Innovation & Precision Dentistry School of Dental Medicine School of Engineering and Applied Sciences University of Pennsylvania Philadelphia Pennsylvania USA; ^4^ The Penn Center for Musculoskeletal Disorders School of Medicine University of Pennsylvania Philadelphia Pennsylvania USA

**Keywords:** bone, Glut1, osteosarcoma, Rb1, Trp53, YAP

## Abstract

Glucose metabolism reprogramming is a critical factor in the progression of multiple cancers and is directly regulated by many tumor suppressors. However, how glucose metabolism regulates osteosarcoma development and progression is largely unknown. Cathepsin K (Ctsk) has been reported to express in chondroprogenitor cells and stem cells besides osteoclasts. Moreover, mutations in the tumor suppressors transformation‐related protein 53 (Trp53) and retinoblastoma protein (Rb1) are evident in approximately 50%–70% of human osteosarcoma. To understand how deletion of Trp53 and Rb1 in Ctsk‐expressing cells drives tumorigenesis, we generated the Ctsk‐Cre;Trp53^f/f^/Rb1^f/f^ mouse model. Our data revealed that those mice developed osteosarcoma without formation of tumor in osteoclast lineage. The level of cortical bone destruction was gradually increased in parallel to the osteosarcoma progression rate. Through mechanistic studies, we found that loss of Trp53/Rb1 in Ctsk‐expressing cells significantly elevated Yes‐associated protein (YAP) expression and activity. YAP/TEAD1 complex binds to the glucose transporter 1 (*Glut1*) promoter to upregulate Glut1 expression. Upregulated *Glut1* expression led to overactive glucose metabolism, increasing osteosarcoma progression. Ablation of YAP signaling inhibited energy metabolism and delayed osteosarcoma progression in Ctsk‐Cre;Trp53^f/f^/Rb1^f/f^ mice. Collectively, these findings provide proof of principle that inhibition of YAP activity may be a potential strategy for osteosarcoma treatment.

## 1 INTRODUCTION

Osteosarcoma is a high‐grade primary malignancy in bone characterized by the deposition of an immature osteoid matrix and new bone by spindle cell tumors.^1^, ^2^, ^3^ Although the annual incidence rate in the United States is only 3.1 cases per million people, osteosarcoma is the most common primary malignancy of bone in children and adolescents after lymphoma and leukemia.^4^, ^5^ Moreover, although osteosarcoma generally shows bimodality across age categories, it can develop at any age. An initial peak is found during the pubertal growth spurt, usually between the ages of 10 and 14 years, and the second peak is observed in adults 60 years of age or older.^4^ Additionally, osteosarcoma is a highly invasive tumor with a metastatic rate of ∼20%, and the most common target for metastasis is the lung.^1^, ^4^, ^6^ Currently, a combination of surgery and chemotherapy is the primary treatment in the clinic. Surgical procedures include amputation of the limb or limb salvage based on the osteosarcoma stage. Early metastasis usually leads to treatment failure and mortality, and the 5‐year survival rate is ∼15%.^1^ Although clear progress has been made through sophisticated surgeries and chemotherapy, limited knowledge of the molecular mechanisms and origin of osteosarcoma pathogenesis has prohibited the development of targeted therapeutics. As a result, clinical outcomes remain poor. Therefore, elucidating the pathological mechanisms of osteosarcoma initiation, development, and metastasis progression is urgently needed for effective prevention, diagnosis, and treatment of osteosarcoma.

Accumulating evidence indicates that mutations of the tumor suppressor genes Trp53 and Rb1 are evident in approximately 50%–70% of human osteosarcoma cases.^6^, ^7^, ^8^ Based on these observations, some mouse osteosarcoma models were generated by mutation of Trp53 and/or Rb1. Mice with conditional double deletions of Trp53 and Rb1 in early limb bud mesenchymal cells induced through Prx1‐Cre displayed osteosarcoma and hibernoma, as well as rhabdomyosarcoma, and deletion of Trp53 alone could also cause osteosarcoma.^7^, ^9^ Moreover, deletion of Trp53 or Trp53 with Rb1 in osteoblast precursors induced by OSX‐Cre led to spontaneous osteosarcoma at a high frequency.^10^ Additionally, osteosarcoma has been also observed in osteoblast‐committed cells when Trp53 and Rb1 are deleted through the use of Col1a1‐Cre and OCN‐Cre transgenic lines,^9^ suggesting that mesenchymal cells and osteogenic lineage cells may be critical for osteosarcoma formation. It is noteworthy that Trp53/Rb1 deficiency in mesenchymal cells mainly results in over 90% of hibernomas instead of osteosarcoma.^7^ Ablation of Trp53/Rb1 in osteoblast lineage cells could develop in the bones anywhere in the body.^10^ However, most of human osteosarcoma occur at the femurs and tibiae around knees. Obviously, these osteosarcoma models could partly mimic human osteosarcoma initiation and progression. Recently, Ctsk has been reported to express in chondroprogenitor cells and periosteal stem cells besides osteoclasts.^11^, ^12^ However, the mutations of Trp53 or Rb1 in Ctsk‐expressing cells that contribute to osteosarcoma formation and pathogenesis are completely unknown.

Hippo pathway is crucial for skeletal development and tumorigenesis through modulating the activity of the core component YAP.^1^, ^13^, ^14^ The enhanced expression and nuclear localization, and decreased phosphorylation of YAP are frequently observed in many types of tumors and is also considered a novel prognostic marker and therapeutic target in osteosarcoma.^1^, ^13^, ^14^ Altered cellular energy metabolism reprogramming is emerging as a key hallmark of tumor cells and malignant transformation and one of the key initiating events in carcinogenesis.^15^, ^16^, ^17^ Glucose is a major source of energy in mammalian cells because it generates ATP through intermediate glycolytic metabolites.^16^, ^18^ In tumor cells, glucose uptake is dramatically increased by the upregulation of glycolytic enzymes such as hexokinase 2 (HK2), 6‐phosphofructo‐2‐kinase/fructose‐2,6‐biphosphatase 3/4 (Pfkfb3/4), lactate dehydrogenase A (LDHA) and the expression of glucose transporters (Gluts, particularly Glut1) to fuel aerobic glycolysis and provide cellular metabolites for the generation of new biomass, which ultimately stimulates tumor development and progression.^19^, ^20^ Recent findings have revealed that suppression of glucose uptake or glycolysis results in a drastic loss of YAP and a reduction in TEA domain transcription factor (TEAD) binding to form complexes, blocking the transcriptional activity of YAP.^21^ Studies have reported that YAP is involved in the regulation of glycolysis in breast cancer^22^ and is an upstream regulator of Glut1 in gastric cancer.^23^ Interestingly, Trp53 and Rb1 signaling has been shown to inhibit YAP activity in tumors such as pancreatic cancer and retinoblastoma,^24^, ^25^ highlighting the crucial role of Trp53 and Rb1 expression as a barrier to tumor progression. Additionally, we have recently demonstrated that YAP governs osteosarcoma progression and lung metastasis.^1^ However, the precise mechanism and pathogenesis for YAP‐mediated glucose metabolism in osteosarcoma caused by Trp53/Rb1 mutations in Ctsk‐expressing cells remains undefined.

In this study, we explored the function and molecular mechanisms by which Trp53/Rb1in Ctsk‐expressing cells drive osteosarcoma formation and progression. Our data showed that deletion of Trp53 and/or Rb1 in Ctsk‐expressing cells activated YAP‐mediated Glut1 expression and energy metabolism and enhanced osteogenic function. These findings provide proof of principle showing that inhibitors of YAP activity may be potential osteosarcoma therapeutics.

## 2 RESULTS

### 2.1 Deletion of Trp53 and Rb1 in Ctsk‐expressing cells causes osteosarcoma formation

**FIGURE 1:**
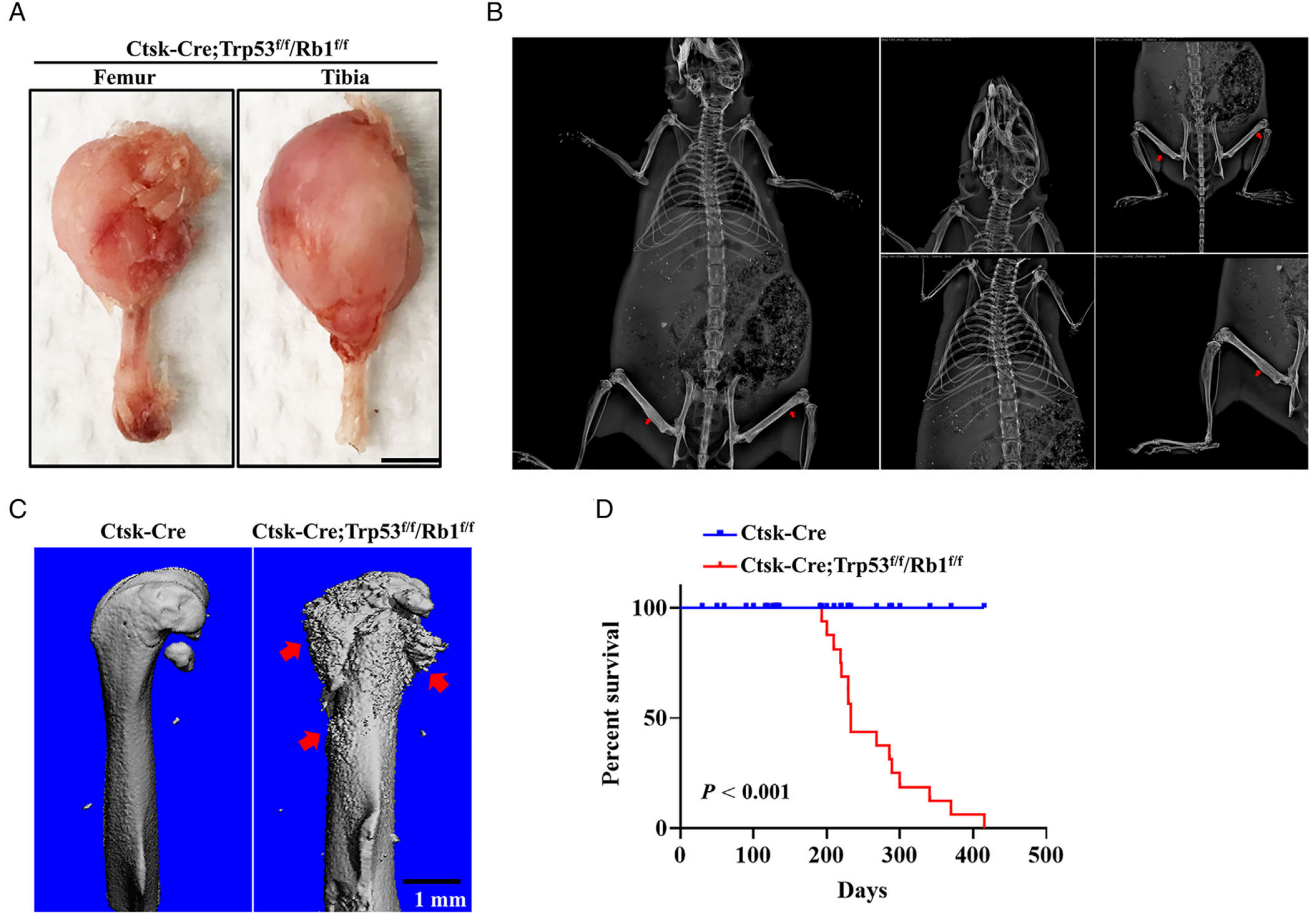
Deletion of Trp53 and Rb1 in Ctsk‐expressing cells drives osteosarcoma formation. (A) Representative osteosarcoma images of femur and tibia from the Ctsk‐Cre;Trp53^f/f^/Rb1^f/f^ mice at age of 7 months. Scale bar: 1 cm. (B) Representative X‐ray images of Ctsk‐Cre;Trp53^f/f^/Rb1^f/f^ mice at age of 6 months. The red arrow indicates the tumor in the bone. (C) Representative micro‐CT images of femurs from the Ctsk‐Cre;Trp53^f/f^/Rb1^f/f^ mice and controls at age of 7 months. The red arrow indicates osteosarcoma in the bone. Scale bar: 1 mm. (D) Kaplan–Meier survival analysis indicating overall survival of Ctsk‐Cre;Trp53^f/f^/Rb1^f/f^ mice and controls

To determine the role of Trp53 and Rb1 in Ctsk‐expressing cells, we first generated a conditional knockout (cKO) line (hereafter named Ctsk‐Cre;Trp53^f/f^/Rb1^f/f^) in which Trp53 and Rb1 were deleted in Ctsk‐expressing cells by crossing Trp53^f/f^/Rb1^f/f^ mice with a transgenic Cre line driven by a *Ctsk* promoter (Ctsk‐Cre). qPCR data verified that Trp53 and Rb1 were largely abrogated in Ctsk^+^ and osteoclast cells (Figure S1A,B). We found that deletion of Trp53 and Rb1 in Ctsk‐expressing cells in mice caused osteosarcoma formation (Figure 1A); however, single deletion of either Trp53 or Rb1 in Ctsk‐expressing cells (Ctsk‐Cre;Trp53^f/f^ and Ctsk‐Cre;Rb1^f/f^) did not lead to osteosarcoma formation at 2, 14, or 18 months (Figure S1C). X‐ray results further showed that no osteosarcoma was formed in other bones, including the calvarium, vertebrae, rib, or sternum; that is, it was found only in the tibia and femur (Figure 1B). Microcomputed tomography (micro‐CT) analysis showed extensive osteosarcoma with sclerosis and cortical destruction in the femurs of 7‐month‐old Ctsk‐Cre;Trp53^f/f^/Rb1^f/f^ mice compared to that in the Ctsk‐Cre control mice (Figure 1C). The Kaplan–Meier survival curves demonstrated a significantly shorter survival rate in the Ctsk‐Cre;Trp53^f/f^/Rb1^f/f^ mice than in the Ctsk‐Cre control mice (Figure 1D).

### 2.2 Deletion of Trp53 and Rb1 in Ctsk‐expressing cells displays a higher osteoblast differentiation ability

**FIGURE 2:**
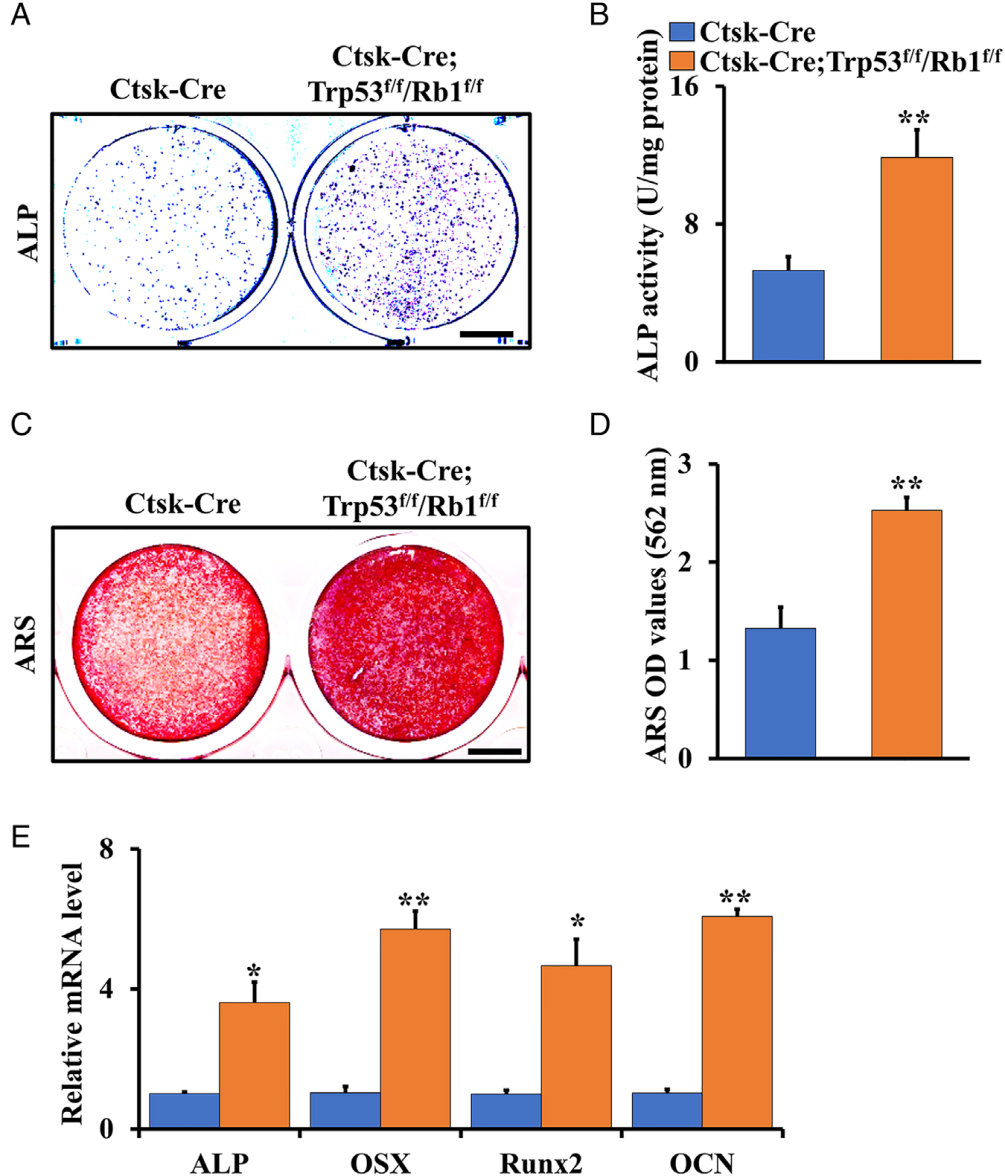
Deletion of Trp53 and Rb1 in Ctsk‐expressing cells displays higher osteoblast differentiation ability. (A) Representative images of ALP (Day 5) staining of Ctsk‐Cre‐positive cells from Ctsk‐Cre;Trp53^f/f^/Rb1^f/f^ and littermate control mice. Scale bar: 1 cm. (B) ALP activity after incubation of osteogenic medium for 7 days. (C) Representative images of ARS (Day 21) staining of Ctsk^+^ cells from Ctsk‐Cre;Trp53^f/f^/Rb1^f/f^ and littermate control mice. (D) Relative OD of (C). (E) qRT‐PCR analysis of the expressions of osteogenic markers as indicated. Error bars are the means ± SEM from three independent experiments. *p < 0.05. **p < 0.01

Han et al. reported that the Ctsk‐Cre‐positive population expanded and filled in the cortical bone of long bone.^11^ To explore the effect of Trp53 and Rb1 deficiency in Ctsk‐expressing cells on activity and differentiation of osteoblasts, we isolated Ctsk^+^ cells from the cortical bone of osteosarcoma area^11^ and explored the function of Trp53 and Rb1 in these cells in vitro by performing alkaline phosphatase (ALP) staining, ALP activity assays, and alizarin red S (ARS) staining. The results showed that loss of Trp53 and Rb1 in Ctsk‐expressing cells significantly enhanced osteoblast differentiation and mineralization (Figure 2A–D). Furthermore, the expression of osteogenic markers was remarkably increased (Figure 2E). Thus, these data indicated that deletion of Trp53 and Rb1 in Ctsk‐expressing cells promoted bone formation.

### 2.3 Osteosarcoma in Ctsk‐Cre;Trp53^f/f^/Rb1^f/f^ mice originates from mesenchymal cells

**FIGURE 3:**
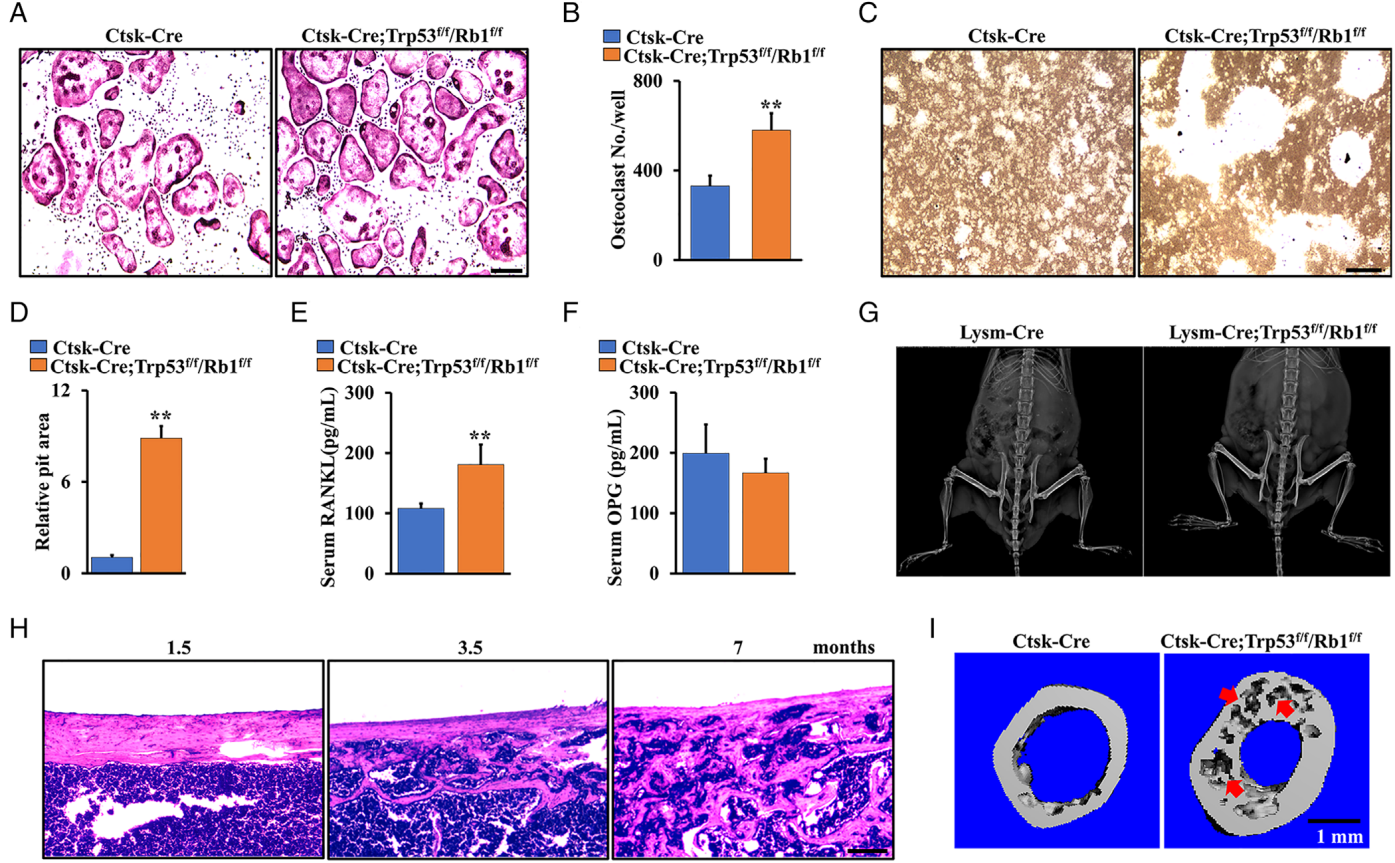
Osteosarcoma in Ctsk‐Cre;Trp53^f/f^/Rb1^f/f^ mice originates from mesenchymal cells. (A) Representative TRAP‐stained images. Monocytes were isolated from Ctsk‐Cre;Trp53^f/f^/Rb1^f/f^ mice and controls, and cultured in the presence of M‐CSF and RANKL. After incubation of 5 days, the cells were stained for TRAP. Scale bar: 100 μm. (B) The corresponding quantitative analysis of (A) was performed. (C and D) Pit formation was analyzed and quantified as indicated. Scale bar: 100 μm. (E and F) Serum levels of RANKL and OPG shown at 4‐month‐old mice. (G) Representative X‐ray images of Lysm‐Cre;Trp53^f/f^/Rb1^f/f^ mice and controls at age of 6 months. (H) Representative H&E‐stained images of cortical bone from Ctsk‐Cre;Trp53^f/f^/Rb1^f/f^ mice and controls as indicated time point. Scale bar: 100 μm. (I) Representative micro‐CT images of cortical bone from the Ctsk‐Cre;Trp53^f/f^/Rb1^f/f^ mice and controls at age of 7 months. The red arrow indicates osteosarcoma in the bone. Scale bar: 1 mm. Error bars are the means ± SEM from three independent experiments. **p < 0.01

Accumulating evidence indicates that Ctsk is a marker of mature osteoclasts and mesenchymal cells.^11^, ^12^, ^26^, ^27^ To identify the effect of Trp53 and Rb1 mutations in Ctsk^+^ cells on osteosarcoma formation, we analyzed the osteoclast number in these cells by tartrate‐resistant acid phosphatase (TRAP) staining. Bone marrow monocytes (BMMs) from Ctsk‐Cre;Trp53^f/f^/Rb1^f/f^ mice and controls were induced with macrophage colony‐stimulating factor (M‐CSF) and receptor activator of nuclear factor kappa‐Β ligand (RANKL) for 5 days for TRAP staining. We found the osteoclast numbers were increased (Figure 3A,B), suggesting that the increased bone formation of Ctsk‐Cre;Trp53^f/f^/Rb1^f/f^ mice did not directly result from impaired osteoclast ability, as evidenced by the analysis of pit formation, serum levels of RANKL, and osteoprotegerin (OPG) (Figure 3C–F). To further confirm this conclusion, we also generated Lysm‐Cre;Trp53^f/f^/Rb1^f/f^ mice using Lysm‐Cre in which the monocytes, macrophages, and osteoclast precursors were deactivated. As expected, X‐ray analysis showed there was no osteosarcoma formation in both tibia and femur at age of 6 months (Figure 3G). These data indicated that osteosarcoma in Ctsk‐Cre;Trp53^f/f^/Rb1^f/f^ mice may have originated from mesenchymal cells. Moreover, we identified the cortical bone architecture of Ctsk‐Cre;Trp53^f/f^/Rb1^f/f^ mice at 1.5, 3.5, and 7 months and found that the thickness and destruction of cortical bone were gradually aggravated, parallel to the progression level of osteosarcoma (Figure 3H). Micro‐CT results also showed apparent bone erosion in the femurs of Ctsk‐Cre;Trp53^f/f^/Rb1^f/f^ mice compared to Ctsk‐Cre mice (Figure 3I).

### 2.4 Loss of Trp53 and Rb1 promotes osteosarcoma progression by overactivating energy metabolism

**FIGURE 4:**
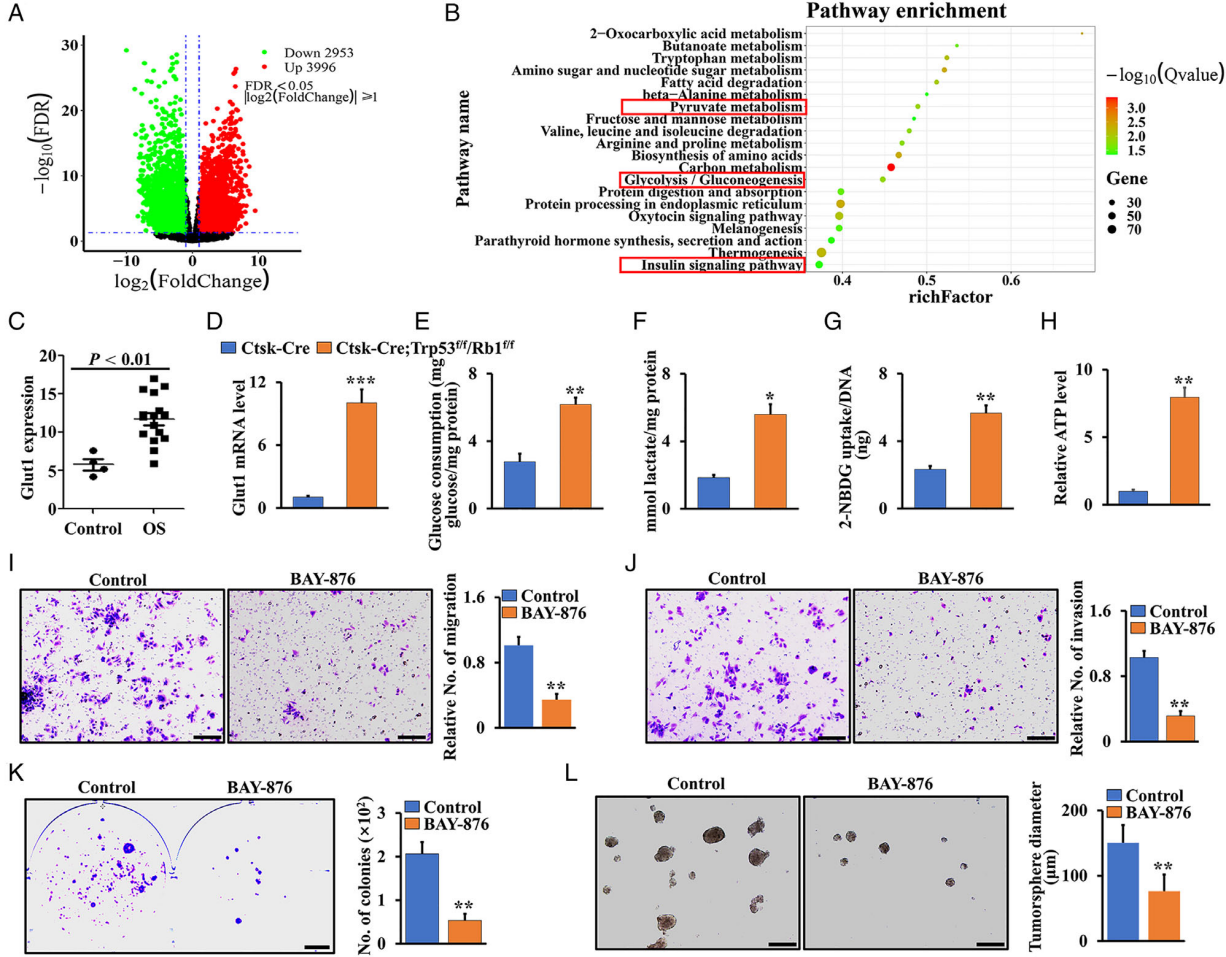
Loss of Trp53 and Rb1 promotes osteosarcoma progression by overactivating energy metabolism. (A) Volcano plot of transcriptome profiles between human osteosarcoma samples and controls from BioProject database under the accession code: PRJNA539828; 3996 genes upregulated; 2953 genes downregulated. (B) KEGG analyses of the osteosarcoma RNA‐seq data showing the top 20 enriched metabolism‐related pathways. Red boxes direct to glucose‐involved metabolic pathways. (C) Glut1 expression between human osteosarcoma samples and controls (BioProject database, PRJNA539828). OS: osteosarcoma. (D) Glut1 expression in the cortical bones of the Ctsk‐Cre;Trp53^f/f^/Rb1^f/f^ mice and controls as indicated. (E–H) The cortical bone cells from Ctsk‐Cre;Trp53^f/f^/Rb1^f/f^ mice and controls as indicated are seeded in 12‐well plate. After incubation for 48 h, the glucose consumption (E), lactate production (F), and ATP level (H) were identified as described in Materials and Methods section. After incubation with 100 μM 2‐NBDG of 8 h, the glucose uptake was determined by a fluorescence microscope at 485/540 nm (G). (I–L) Analyses of the cortical bone cells from Ctsk‐Cre;Trp53^f/f^/Rb1^f/f^ mice treated with or without 1 μM Glut1 inhibitor Bay‐876 as indicated. The corresponding quantification was identified at right. (I) Migration. (J) Invasion. (K) Soft agar. (L) Tumorsphere. Scale bar: 100 μm. Error bars are the means ± SEM from three independent experiments. *p < 0.05, **p < 0.01, ***p < 0.001

To further define the mechanisms by which Trp53/Rb1 regulates osteosarcoma progression, we analyzed publicly available human osteosarcoma data in the BioProject database (ID: PRJNA539828).^2^ A volcano plot of the data showed a set of significantly downregulated (2953) and upregulated (3996) genes with more than two‐fold change compared to their expression in normal tissues (Figure 4A). Interestingly, further analysis of these upregulated genes showed that glucose‐involved metabolism is a conserved signature of one of the most significantly enriched gene sets (Figure 4B). Recent evidence suggests that Glut1 is the dominant glucose transporter during glucose metabolism in both cancer and osteoblast differentiation.^28^, ^29^, ^30^, ^31^ To further investigate how Trp53 and Rb1 affect glucose metabolism in osteosarcoma, we first analyzed Glut1 expression reported in the database (ID: PRJNA539828).^2^ The results showed that Glut1 expression was increased in human osteosarcoma tissues compared to control tissues (Figure 4C). Consistently, assessing the osteosarcoma tissue of Ctsk‐Cre;Trp53^f/f^/Rb1^f/f^ mice, we found highly increased Glut1 expression compared to that in normal cortical bone (Figure 4D). To gain further insights into the regulation of Trp53/Rb1‐mediated glucose metabolism in osteosarcoma, we further assessed glucose consumption, lactate production, 2‐NBDG uptake, and ATP levels using cortical bone cells from Ctsk‐Cre;Trp53^f/f^/Rb1^f/f^ mice and controls. Interestingly, we found that these indices of glucose‐related metabolism were significantly increased in the Ctsk‐Cre;Trp53^f/f^/Rb1^f/f^ group compared to those in the control group (Figure 4E–H). To further examine whether inhibition of Glut1 suppresses osteosarcoma, cell migration and invasion assays were performed in primary osteosarcoma cells derived from Ctsk‐Cre;Trp53^f/f^/Rb1^f/f^ mice with or without treatment with the Glut1 inhibitor BAY‐876 for 48 h. The results showed that cell migration and invasion were significantly inhibited after BAY‐867 treatment (Figure 4I,J). Notably, we also found that the colony‐formation ability (Figure 4K) and tumorsphere diameter (Figure 4L) were significantly decreased after treatment with BAY‐876 for 48 h.

### 2.5 Trp53 and Rb1 orchestrate energy metabolism in osteosarcoma

**FIGURE 5:**
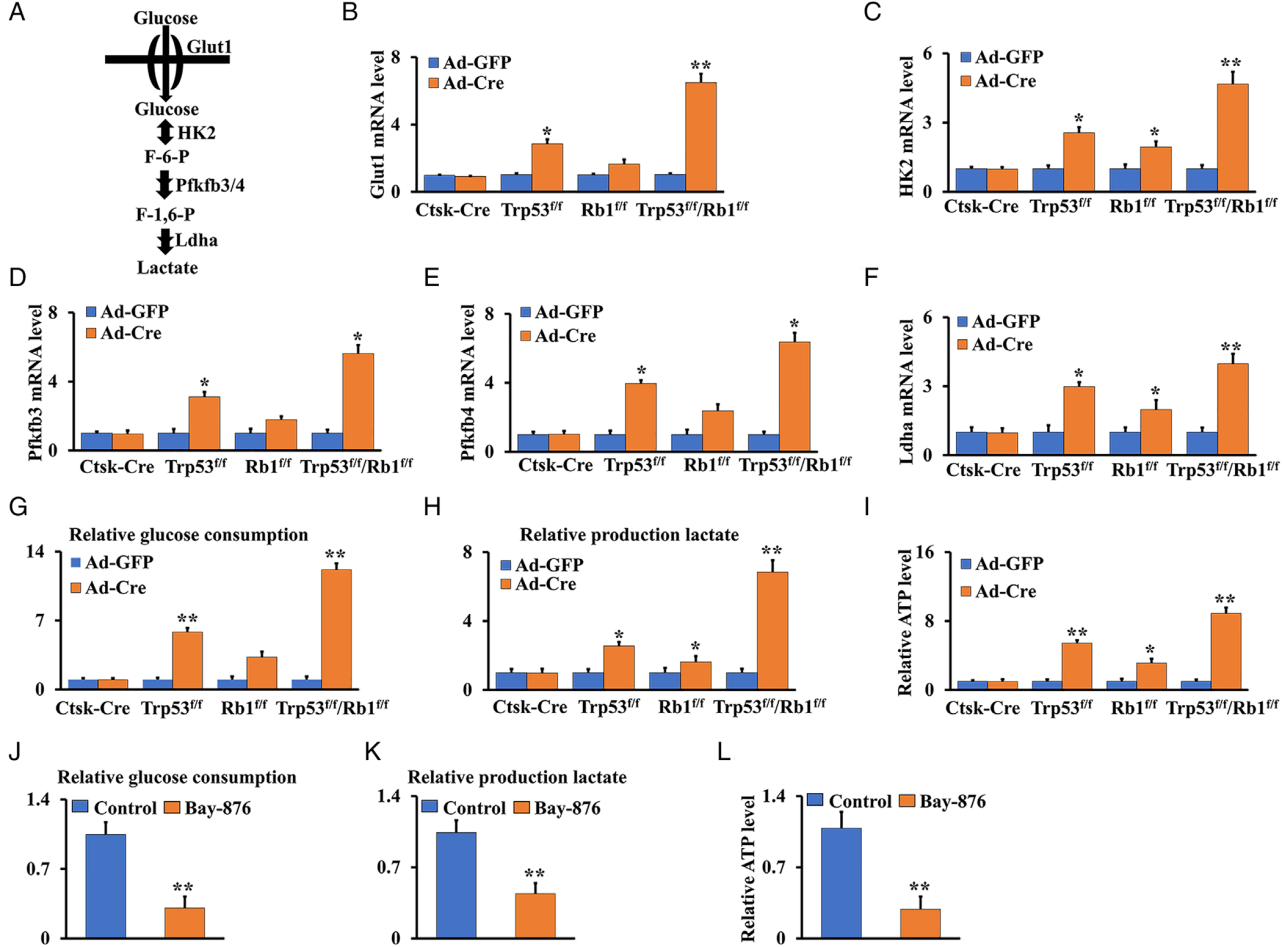
Trp53 and Rb1 orchestrate energy metabolism through Glut1 in osteosarcoma. (A) Diagram of the key enzymes in the glucose metabolism such as Glut1, Hk2, Pfkfb3/4 and Ldha. (B–F) mRNA expressions of Glut1, Hk2, Pfkfb3/4 and Ldha were identified after transfection for 48 h with Ad‐Cre or Ad‐GFP in the cortical bone cells from Trp53^f/f^/Rb1^f/f^ mice and controls as indicated. (G–I) The cortical bone cells from Ctsk‐Cre;Trp53^f/f^/Rb1^f/f^ mice and controls as indicated are seeded in 12‐well plate. After incubation for 48 h, the glucose consumption (G), lactate production (H), and ATP level (I) were identified as described in Materials and Methods section. (J–L) The glucose consumption (J), lactate production (K), and ATP level (L) were identified in cortical bone cells from Ctsk‐Cre;Trp53^f/f^/Rb1^f/f^ mice after treatment of 48 h with or without 1 μM Glut1 inhibitor Bay‐876. Error bars are the means ± SEM from three independent experiments. *p < 0.05, **p < 0.01

To further understand the regulation of Trp53 and Rb1 in glucose metabolism, primary cortical bone cells were isolated from Trp53^f/f^/Rb1^f/f^ and control mice, and infected with adenoviruses expressing Cre (Ad‐Cre) or control (Ad‐GFP) in vitro. We first confirmed the effective deletion of Trp53 and Rb1 in Ad‐Cre‐treated cells by qPCR analysis (Figure S2A,B). Then, the infected cells were used to identify the expression of key genes in glucose metabolism, including *Glut1*, *HK2*, *Pfkfb3/4*, and *Ldha* (Figure 5A). As expected, the expression of these glucose metabolism‐related genes was significantly upregulated in Trp53‐/Rb1‐deficient cells compared to that in the control after Ad‐GFP infection (Figure 5B–F). The values of glucose consumption and lactate production in single Trp53‐ or Rb1‐deficient cells were approximately 5.83/3.28‐fold and 2.55/1.62‐fold increased, respectively, compared to those in the control cells without Trp53 or Rb1 deletion; however, these indices from double Trp53/Rb1‐deficient cells reached approximately 12.45‐fold and 6.84‐fold, respectively (Figure 5G,H), suggesting that double deletion of Trp53 and Rb1 significantly enhanced energy metabolism, as evidenced by increased ATP levels in Trp53‐ and/or Rb1‐deficient cells (Figure 5I). To further investigate Glut1 function in metabolic regulation by Trp53 and Rb1, osteosarcoma cells from Ctsk‐Cre;Trp53^f/f^/Rb1^f/f^ mice were treated with the Glut1 inhibitor BAY‐876 for 48 h. The results showed that glucose consumption, lactate production, and ATP levels in the cells were significantly reduced (Figure 5J–L). These results indicated that Trp53 and Rb1 orchestrated glucose‐mediated energy metabolism in osteosarcoma.

### 2.6 Enhanced energy metabolism in osteosarcoma depended on increased YAP activity and YAP‐mediated Glut1 expression

**FIGURE 6:**
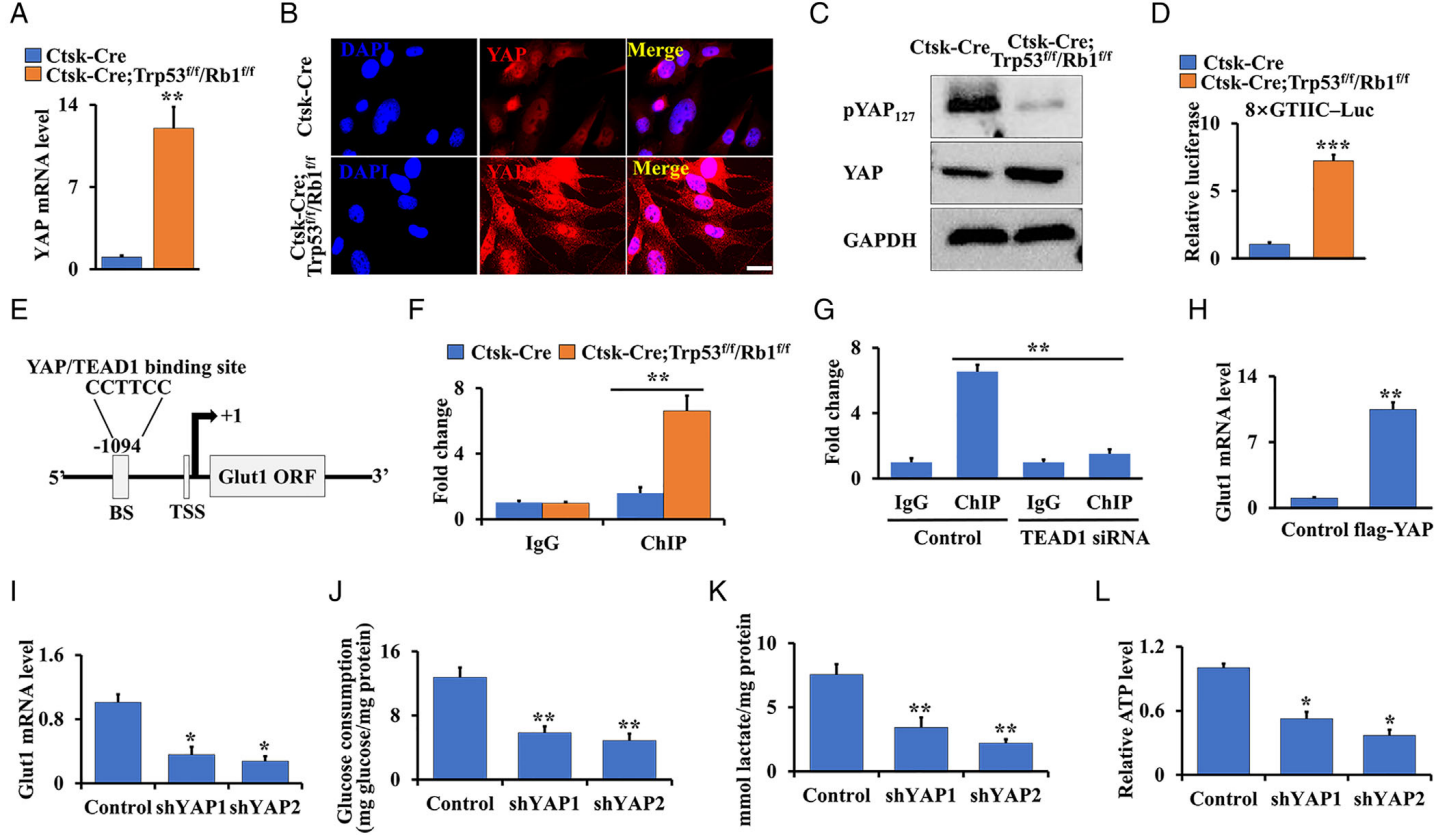
Enhanced energy metabolism in osteosarcoma depends on increased YAP activity and its mediated‐Glut1 expression. (A) qRT‐PCR analysis of YAP in cortical bone of Ctsk‐Cre;Trp53^f/f^/Rb1^f/f^ mice and controls. (B) Representative images of immunofluorescent staining of YAP in the cortical bone cells from Ctsk‐Cre;Trp53^f/f^/Rb1^f/f^ mice and controls. Scale bars: 25 μm. (C) Western blot as indicated. (D) The cortical bone cells from Ctsk‐Cre;Trp53^f/f^/Rb1^f/f^ mice and controls were co‐transfected with luciferase reporter and pRL‐TK plasmids (internal control) as indicated, respectively. After transfection of 48 h, the luciferase activities were identified by the Dual‐Luciferase Assay Kit. (E) Schematic binding diagram of YAP/TEAD1 complex in *Glut1* promoter. BS and TSS represented binding site and transcription start site, respectively. (F and G) ChIP assay. Co‐occupation of YAP/TEAD1 complex in *Glut1* promoter in cortical bone cells from Ctsk‐Cre;Trp53^f/f^/Rb1^f/f^ mice and controls (F). Co‐occupation of YAP/TEAD1 in *Glut1* promoter after silence of *TEAD1* (G). (H and I) qRT‐PCR analysis of Glut1 after overexpression (H) or silence (I) of YAP. (J–L) After silence of YAP in cortical bone cells from Ctsk‐Cre;Trp53^f/f^/Rb1^f/f^ mice for 48 h, the glucose consumption (J), lactate production (K), and ATP level (L) were identified as described in Materials and Methods section. Error bars are the means ± SEM from three independent experiments. *p < 0.05, **p < 0.01, ***p < 0.001

Trp53/Rb1 have been proven to be involved in the progression of many tumors by activating YAP.^24^, ^25^ Our previous study showed that YAP expression and activity are increased in osteosarcoma, and the development and metastasis of osteosarcoma are governed by the YAP/TEAD1 complex.^1^ Moreover, Glut1‐mediated glucose metabolism plays an important role in tumor progression and plasticity.^15^ To further test whether abnormal energy metabolism in Trp53/Rb1 mutant osteosarcoma is associated with YAP signaling, the expression and activity of YAP were first identified in cortical bone cells from Ctsk‐Cre;Trp53^f/f^/Rb1^f/f^ mice and controls. Interestingly, loss of Trp53 and Rb1 in Ctsk‐expressing cells significantly increased YAP expression and nuclear translocation (Figure 6A,B), suggesting YAP signaling was enhanced in these mutant cells, as evidenced by the analysis of YAP target genes’ expression (Figure S3) and its phosphorylation (Figure 6C). Consistently, luciferase assays showed that YAP/TEAD1 transcriptional activity was also significantly increased (Figure 6D). Given that activation of YAP transcriptionally controls target gene expression,^1^, ^13^, ^14^ to further determine whether YAP regulates Glut1 transcriptional expression, we analyzed the motif to which the YAP/TEAD1 complex binds the promoter region of the *Glut1* gene using Vector NTI software. Interestingly, we found a binding site for the YAP/TEAD1 complex located from –1094 to –1100 base pairs in the region of the *Glut1* promoter (Figure 6E). Next, ChIP assay analysis of the cortical bone cells from Ctsk‐Cre;Trp53^f/f^/Rb1^f/f^ mice and controls showed a clear increase in the transcriptional activity of Glut1 in the control cells (Figure 6F); however, the activity was inhibited after *TEAD1* expression was silenced by siRNA (Figure 6G; Figure S2C). Furthermore, we found that forced expression of YAP promoted Glut1 expression, which was inhibited by silencing YAP (Figure 6H,I; Figure S2D,E). To verify the potential role of YAP in glucose metabolism, we next explored glucose consumption, lactate production, and ATP levels. Intriguingly, we found that glucose consumption, lactate production, and ATP levels decreased by 2.18‐/2.62‐, 2.21‐/3.42‐, and 1.9‐/2.72‐fold, respectively, in the shYAP1 and shYAP2 groups compared to the control cells, demonstrating that silencing YAP inhibited glucose metabolism (Figure 6J–L). Taken together, these data indicated that enhanced Glut1‐mediated energy metabolism depends on increased YAP activity in osteosarcoma.

### 2.7 Ablation of YAP inhibited osteosarcoma progression in Ctsk‐Cre;Trp53^f/f^/Rb1^f/f^ mice

**FIGURE 7:**
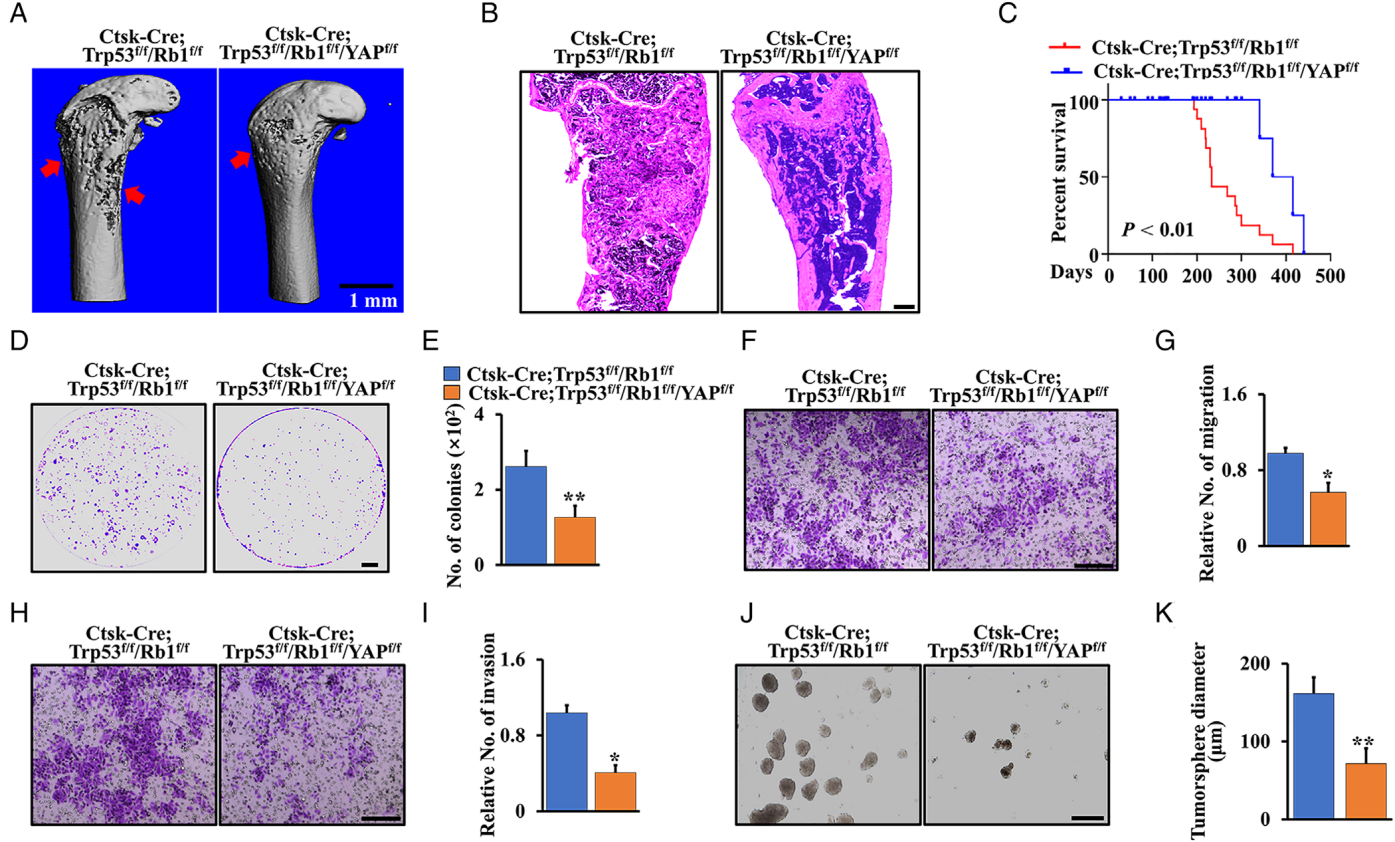
Ablation of YAP inhibited the osteosarcoma progression in Ctsk‐Cre;Trp53^f/f^/Rb1^f/f^ mice. (A) Representative micro‐CT images of femurs of Ctsk‐Cre;Trp53^f/f^/Rb1^f/f^ and Ctsk‐Cre;Trp53^f/f^/Rb1^f/f^/YAP^f/f^ mice at 6 months. Scale bars: 1 mm. (B) Representative H&E‐stained images of femur sections from Ctsk‐Cre;Trp53^f/f^/Rb1^f/f^ and Ctsk‐Cre;Trp53^f/f^/Rb1^f/f^/YAP^f/f^ mice at 6 months. Scale bar: 1 mm. (C) Kaplan–Meier survival analysis indicating overall survival of Ctsk‐Cre;Trp53^f/f^/Rb1^f/f^ and Ctsk‐Cre;Trp53^f/f^/Rb1^f/f^/YAP^f/f^ mice. (D and E) The crystal violet staining (D) and analysis (E) of soft agar using cortical bone cells from Ctsk‐Cre;Trp53^f/f^/Rb1^f/f^ and Ctsk‐Cre;Trp53^f/f^/Rb1^f/f^/YAP^f/f^ mice. (F–I) Cell migration (F), invasion (H), and corresponding quantification (G and I). (J) Tumorsphere. Scale bar: 100 μm. (K) The quantification of tumorsphere diameter was identified from (J). Error bars are the means ± SEM from three independent experiments. *p < 0.05, **p < 0.01

To further assess the role of YAP in glucose metabolism and osteosarcoma progression in Ctsk‐Cre;Trp53^f/f^/Rb1^f/f^ mice, we deleted YAP in Ctsk‐Cre;Trp53^f/f^/Rb1^f/f^ mice to generate a triple‐cKO mouse model, Ctsk‐Cre;Trp53^f/f^/Rb1^f/f^/YAP^f/f^ (triple cKO). As expected, micro‐CT data showed that ablation of YAP protected against bone erosion in Ctsk‐Cre;Trp53^f/f^/Rb1^f/f^ (double cKO) mice (Figure 7A). H&E staining of femurs from 6‐month‐old triple‐cKO mice also showed much less expansive osteosarcoma osteoid lesions in the cortical bone compared to that in Ctsk‐Cre;Trp53^f/f^/Rb1^f/f^ mice (Figure 7B), indicating that inactivation of YAP blocked osteosarcoma progression in the Ctsk‐Cre;Trp53^f/f^/Rb1^f/f^ mice. Additionally, the Kaplan–Meier survival curves plotted for the mice showed a significantly longer mean survival rate in the triple‐cKO mice than in the double‐cKO mice (Figure 7C). To further test whether loss of YAP inhibits tumor cell growth, we performed WST‐1 and soft agar assays using cortical bone cells from triple‐cKO and double‐cKO mice. Notably, tumor cell growth was significantly inhibited after the loss of YAP (Figure 7D,E; Figure S4). Moreover, deletion of YAP significantly inhibited cell migration and invasion (Figure 7F–H). In accordance with the reduced mobility of osteosarcoma cells, loss of YAP remarkably decreased the numbers and size of tumorspheres compared with those in the double‐cKO group (Figure 7J,K).

## 3 DISCUSSION

**FIGURE 8:**
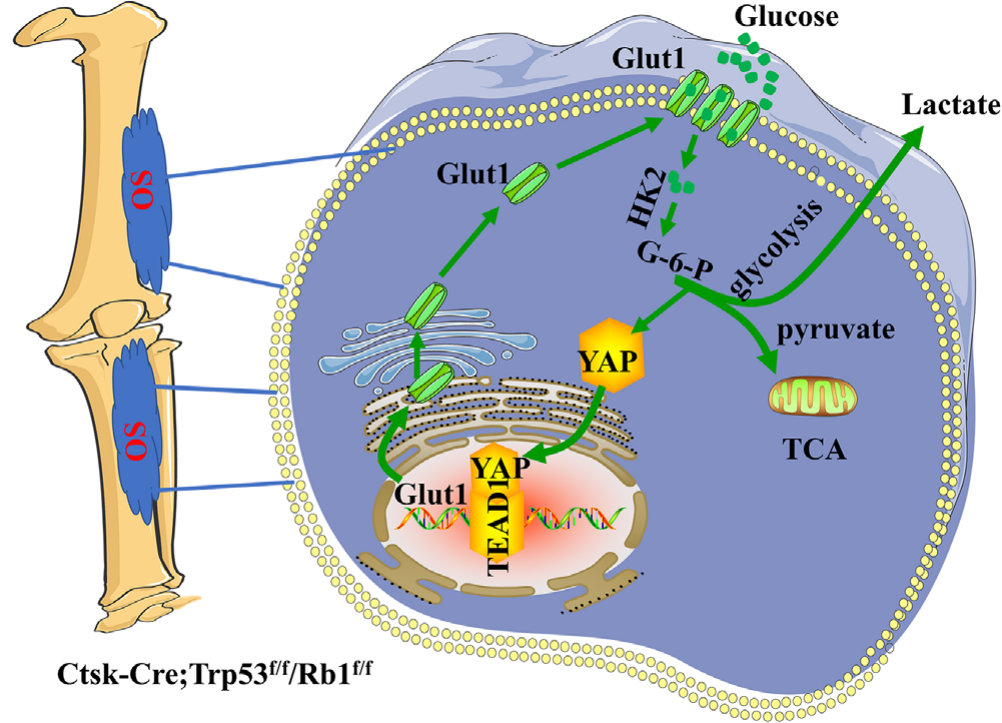
Deletion of Trp53 and Rb1 in Ctsk‐expressing cells drives osteosarcoma progression by activating YAP‐mediated energy metabolism. Glut1‐mediated glucose metabolism was enhanced after loss of Trp53 and Rb1 in Ctsk‐expressing cells, which was dependent on YAP activity and directly regulated by YAP/TEAD1 complex

Studies have shown that Trp53 and Rb1 play critical roles in bone development, remodeling, and tumorigenesis.^7^, ^32^, ^33^, ^34^, ^35^ Deletion of Trp53 and Rb1 in Prx1‐positive mesenchymal lineage cells causes osteosarcoma^7^; consistent with this previous finding, other studies have shown osteosarcoma development after loss of Trp53 and Rb1 in osteoblast precursors and osteocytes, indicating that osteogenic lineage cells may be the principal sources for osteosarcoma formation.^8^, ^9^, ^10^ Accumulating evidence indicates that Ctsk is a marker of chondroprogenitor cells and mesenchymal cells in addition to mature osteoclasts.^11^, ^12^, ^26^ In our study, we demonstrated that deletion of Trp53 and Rb1 in Ctsk‐expressing cells drove osteosarcoma formation, and the osteosarcoma in Ctsk‐Cre;Trp53^f/f^/Rb1^f/f^ mice originated from mesenchymal cells of the cortical bone. We further found, for the first time, that loss of Trp53 and Rb1 overactivated YAP in Ctsk‐expressing cells to enhance Glut1 expression and energy metabolism, and thereby promote osteosarcoma progression (Figure 8). Our new findings provide proof of principle that inhibition of YAP activity may be a potential strategy for treatment of osteosarcoma.

Previous evidence indicates that Ctsk^+^ cells are mesenchymal cells that govern intramembranous bone formation and cortical bone repair.^26^, ^27^ On the other hand, Han et al. reported that Ctsk^+^ cells mainly expanded and filled in the cortical bone and that deletion of Lkb1 in Ctsk‐expressing cells induced osteosarcoma by activating mTORC1 signaling.^11^ Consistent with these findings, we found that Trp53/Rb1 deficiency in Ctsk‐expressing cells resulted in osteosarcoma and that the level of cortical bone destruction was gradually aggravated in parallel with the progression rate of osteosarcoma. Moreover, the expression levels of osteogenic markers and activity of osteogenic differentiation were significantly enhanced in cortical bone cells from Ctsk‐Cre;Trp53^f/f^/Rb1^f/f^ mice, suggesting that Ctsk‐expressing cells in the cortical bone are the principal sources of osteosarcoma formation. Recent studies also reported that osteocytes can express and secrete Ctsk.^36^, ^37^ Additionally, osteoblasts and osteocytes are the major cells to produce RANKL and OPG.^38^, ^39^ Therefore, we measured the serum levels of RANKL and OPG in 4‐month‐old Ctsk‐Cre;Trp53^f/f^/Rb1^f/f^ mice and controls. Interestingly, we found serum RANKL showed a significant increase; however, the serum OPG had no significant change. The ratio of RANKL/OPG is much higher in Ctsk‐Cre;Trp53^f/f^/Rb1^f/f^ mice compared to that in the control, suggesting that osteoblasts/osteocytes derived from Ctsk^+^ cells with loss of Trp53 and Rb1 may promote osteoclastogenesis and osteolysis in tumor microenvironment through increasing RANKL expression. Additionally, we found a notable increase in osteoclast numbers after deletion of Trp53 and Rb1 in Ctsk‐expressing cells. More importantly, no osteosarcoma formation was detected after deletion of Trp53 and Rb1 in LysM^+^ cells, which appeared to rule out the likelihood that osteosarcoma formation was directly caused by impaired osteoclastogenesis. Supporting our findings, a previous study has reported that deletion of Lkb1 in Ctsk‐expressing cells caused osteosarcoma formation; in contrast, no osteosarcoma was observed after deletion of Lkb1 using LysM‐Cre in osteoclast precursors.^11^ However, Yang et al. reported that deletion of Ptpn11 in Ctsk‐expressing cells led to metachondromatosis by activating Hedgehog signaling, but LysM‐Cre;Ptpn11^f/f^ mice displayed a mild phenotype of osteopetrosis.^12^ But, how Ctsk‐expressing cells precisely coordinate the function of osteoclasts, chondrocytes, osteoblasts, and osteocytes is largely unknown and needs to be further investigated in the future.

Glucose is a major source of energy in mammalian cells because it upregulates the expression of glycolytic enzymes such as Glut1, HK2, Pfkfb3/4, and LDHA to stimulate tumorigenesis.^16^, ^19^, ^40^, ^41^ Overexpression of Glut1 has been commonly observed in many cancers, such as osteosarcoma, lung cancer, and lymphoma.^41^, ^42^ In addition, Glut1 is considered to be the main isoform expressed in human osteosarcoma cell lines (SaOS2, MG‐63, and U2‐OS cell lines).^43^ Previous studies have also shown that targeting glucose metabolism has the potential to provide an effective approach for cancer therapy.^44^, ^45^ However, therapeutic roles of glucose metabolism in osteosarcoma remain unclear. Interestingly, by analyzing the human osteosarcoma database, we revealed that glucose‐involved metabolism is a conserved signature of one of the most significantly enriched gene sets. More importantly, our data showed that loss of Trp53 and Rb1 in Ctsk‐expressing cells significantly upregulated Glut1 expression and glucose consumption, lactate production, and ATP levels, which were reversed by treatment with the Glut1 inhibitor BAY‐876. Similarly, other studies also demonstrated that Glut1 expression is negatively regulated by Trp53 in SaOS‐2 cells, controlling cell energy supply and glucose metabolism.^46^ Furthermore, inhibition of Trp53 has been shown to enhance Glut1 expression and thus promote glucose metabolism during osteogenesis.^47^ Overall, our findings provide evidence for glucose metabolism as a robust biomarker that can be readily assessed by qRT‐PCR in routine osteosarcoma diagnostics.

YAP signaling is crucial for tumorigenesis and osteogenesis because it regulates cell proliferation, differentiation, and apoptosis.^1^, ^48^ Our previous study demonstrated that YAP regulates osteosarcoma progression and lung metabolism.^1^ Recent studies have highlighted the fact that suppression of glucose uptake or glycolysis resulted in a dramatic loss of YAP/TEAD complex binding, inhibiting the transcriptional activity of YAP.^21^, ^49^, ^50^ Our data showed that Trp53 and Rb1 inhibited YAP expression and activation. Loss of Trp53 and Rb1 in Ctsk‐expressing cells led to increased YAP expression and nuclear translocation and eventually enhanced the transcriptional activity of the YAP/TEAD1 complex. Additionally, our ChIP‐qPCR data demonstrated that Glut1 expression was directly regulated by the YAP/TEAD1 complex, as evidenced by the outcomes of silencing *TEAD1* and forced expression of YAP. However, it is also possible that increased Glut1 expression is partly due to the enhanced expression of YAP. Moreover, we found that silencing YAP significantly inhibited glucose metabolism by decreasing Glut1 expression. Consistently, global knockout of YAP in zebrafish has been shown to cause glucose defects by decreasing Glut1 expression.^51^ In bladder cancer, it has also been found that YAP is involved in glucose metabolism by regulating Glut1 expression,^52^ demonstrating that YAP is an upstream regulator of Glut1. More importantly, we found that loss of Trp53 and Rb1 in Ctsk^+^ cells promoted osteosarcoma cell migration and invasion; however, ablation of YAP signaling delayed osteosarcoma progression in Ctsk‐Cre;Trp53^f/f^/Rb1^f/f^ mice due to the inhibition of migration and invasion. In consistent with our findings, silence of YAP in osteosarcoma cells is also found to prohibit the tumor cell growth and tumor formation.^1^, ^53^ Likewise, our previous findings also showed that inhibition of YAP delays osteosarcoma progression and lung metastasis in mice.^1^

In summary, our findings reveal, for the first time, that Trp53/Rb1 deficiency in Ctsk‐expressing cells causes osteosarcoma. Moreover, YAP‐mediated energy metabolism is involved in Trp53/Rb1‐driven osteosarcoma progression. These findings strengthen the link between tumor formation and cellular metabolism and provide new evidence showing that targeting the YAP‐mediated glucose metabolic pathway may be a promising therapeutic strategy for osteosarcoma and other bone diseases and cancers.

## 4 MATERIALS AND METHODS

### 4.1 Mice

Ctsk‐Cre, Td^+/f^, and YAP^f/f^ mice were purchased from Jackson Laboratory (Bar Harbor, USA). Trp53^f/f^/Rb1^f/f^ mice were a gift from Dr. David M. Feldser's lab at Department of Cancer Biology, University of Pennsylvania.

### 4.2 Antibodies, reagents, plasmids, and transfection

Antibody against pYAP127 (D9W2I), YAP (D8H1X) was purchased from Cell Signaling Technology. The secondary fluorescent antibodies and H&E staining kit were from Abcam. Glut1 inhibitor Bay‐876 was obtained from MCE Med Chem Express. The fluorescent glucose analog 2‐(N‐(7‐nitrobenz‐2‐oxa‐1,3‐diazol‐4‐yl) amino)‐2‐deoxyglucose (2‐NBDG) was from Cayman Chemical Company. *TEAD1* siRNAs were purchased from Santa Cruz Biotechnology. TRAP staining kit was purchased from Sigma. Plasmids pRL‐TK, pcDNA3.1, pcDNA3.1‐flag‐YAP, and shYAP1/2 were obtained from Addgene. The transfection was performed as we previously reported.^1^, ^54^, ^55^

### 4.3 Cell proliferation, migration, invasion, and tumorsphere assays

Cell proliferation, migration, invasion, and tumorsphere assays were carried out as we previously reported.^1^, ^54^

### 4.4 Cell culture and luciferase reporter assay

The cell culture of cortical bone cells was performed as described by Han et al.^11^ Briefly, cortical bone cells were isolated from the femurs of 4‐month‐old Ctsk‐Cre;Trp53^f/f^/Rb1^f/f^, Ctsk‐Cre;Trp53^f/f^/Rb1^f/f^/YAP^f/f^, and Trp53‐ and/or Rb1‐floxed mice. Briefly, fresh femurs were thoroughly cleaned after removing all soft tissue, especially muscles, and then the bone marrow was thoroughly flushed three times with sterile ice‐cold PBS. Next, the diaphyses from the femurs were collected and digested in α‐MEM containing 2 mg/ml collagenase (Sigma, USA) and 2 mg/ml dispase II (Invitrogen, USA) at 37°C for 30 min. Subsequently, after digestion, these cells were harvested and cultured in α‐MEM (Gibco, USA) supplemented with 10% FBS (Gibco, USA) and 1% Pen‐Strep solution (Gibco, USA) at 37°C with 5% CO_2_ under humid conditions. The medium was replaced every other day.

For the luciferase reporter assay, cortical bone cells were seeded and cotransfected with luciferase reporter and the indicated plasmids in a 12‐well plate. After culturing for 48 h, the luciferase activity levels were determined with a Dual‐Luciferase assay kit.^1^

### 4.5 Flow cytometry

To sort Ctsk^+^ cells, we first isolated the primary cortical bone cells from 4‐month‐old Ctsk‐Cre;Trp53^f/f^/Rb1^f/f^/Td^+/f^ mice and controls and cultured the cells for 2 days.^11^ And then the cell sorting was performed after digesting these cells to a single cell suspension. The sorted cells were cultured in α‐MEM supplemented with 10% FBS and 1% Pen‐Strep solution at 37°C with 5% CO_2_ under humid conditions.

### 4.6 qRT‐PCR

Briefly, 1 μg of total RNA extracted from cortical bones of 4‐month‐old Ctsk‐Cre, Ctsk‐Cre;Trp53^f/f^/Rb1^f/f^, and Trp53‐ and/or Rb1‐floxed mice using TRIzol reagent (TaKaRa, Japan) was reverse‐transcribed into cDNA by PCR using a PrimeScript RT Kit (TaKaRa, Japan). Then, qRT‐PCR was performed using a CFX96 Real‐Time PCR System with SYBR Green (Bio‐Rad, USA). GAPDH served as the internal control, and expression was determined by the 2^–∆∆Ct^ method. The qRT‐PCR primers used in this study are listed in Table S1.

### 4.7 ALP staining and ALP activity

For ALP staining, briefly, cortical bone cells from Ctsk‐Cre;Trp53^f/f^/Rb1^f/f^ and Ctsk‐Cre mice (controls) were induced with osteogenic medium (α‐MEM containing 10% FBS, a 1% Pen‐Strep solution, 100 nM dexamethasone [Sigma, USA], 50 μg/ml L‐ascorbic acid [Sigma, USA], and 5 mM β‐glycerophosphate [Sigma, USA]). The osteogenic medium was replaced every 3 days. After 5 days of osteogenic induction, the cells were fixed with 4% paraformaldehyde (PFA) for 30 s at room temperature, and then ALP staining was carried out with a BCIP/NBT ALP staining kit (Millipore, USA) according to the manufacturer's instructions.

After 7 days of osteogenic induction, ALP activity was measured by determining OD_405_ nm with a microplate reader. Briefly, cells were harvested with harvest buffer (2 mM PMSF and 0.2% NP‐40 in 10 mM Tris‐Cl [pH 7.4]) after washing two times with ice‐cold PBS, and then the supernatants were collected and incubated with assay buffer containing 1 mM MgCl_2_, 100 mM glycine (pH 10.5) and 50 mM p‐nitrophenyl phosphate solution for 15 min at 37°C. Subsequently, the reaction was stopped by adding 0.1 N NaOH solution, and the absorbance was measured at OD_405_ nm, and the production of p‐nitrophenol (nmol) in total protein (per minute per mg) was determined as we previously described.^56^

### 4.8 Osteoblast differentiation and alizarin red S staining

Osteogenic differentiation was induced for 3 weeks by osteogenic medium as we described above, and then the cells were stained with alizarin red S staining solution (pH 4.4). After image scanning, the stained cells were thoroughly destained with 10% cetylpyridinium chloride in 10 mM sodium phosphate buffer (pH 7.0), measured and quantified with a microplate reader at OD_562_ nm.^56^

### 4.9 Osteoclast differentiation, TRAP staining, and pit formation

Mouse bone marrow‑derived macrophages (BMMs) were isolated from femurs of 2‐month‐old Ctsk‐Cre;Trp53^f/f^/Rb1^f/f^ and Ctsk‐Cre mice (controls), seeded in a 96‐well plate, and then induced by osteoclastogenic medium containing 100 ng/ml recombinant human RANKL and 20 ng/ml M‐CSF for 5 days.^57^, ^58^ Mature osteoclasts were fixed in 4% PFA and characterized by staining with a TRAP staining kit (Sigma, USA) according to the manufacturer's instructions. The pit formation was performed as we previously reported.^58^

### 4.10 Metabolite measurements

Cortical bone cells were isolated from femurs of 4‐month‐old Ctsk‐Cre;Trp53^f/f^/Rb1^f/f^ mice and controls or Trp53‐ and/or Rb1‐floxed mice as outlined in the cell culture section. For in vivo experiments, we used primary cortical cells from Ctsk‐Cre;Trp53^f/f^/Rb1^f/f^ mice and controls. For in vitro experiments, each cell pool obtained from Trp53‐ and/or Rb1‐floxed mice and controls was split into two sets of cell groups: a control group and an experimental group, and then transduced with adenovirus Ad‐Cre or control Ad‐GFP, respectively. Next, glucose consumption and lactate production were measured with a glucose (HK) assay kit (Sigma, USA) and L‐lactate assay kit (Eton Biosciences, USA), respectively, according to the respective manufacturer's instructions.^19^, ^59^ For the glucose uptake assay, after the cells were incubated with 100 μM 2‐NBDG for 8 h, the glucose uptake was determined with a fluorescence microscope at 485/540 nm. ATP production was quantified using a CellTiter‐Glo Luminescent Cell Viability Assay Kit (Promega, USA).^19^, ^59^ Serum levels of OPG and RANKL were measured by mouse OPG ELISA Kit (Boster Biological Technology) and TNFSF11/RANKL PicoKine ELISA Kit (Boster Biological Technology) according to the manufacturer's instructions.

### 4.11 ChIP‐qPCR

ChIP‐qPCR was performed with an Imprint chromatin immunoprecipitation kit (Sigma, USA) as we previously reported.^1^, ^54^, ^60^ Briefly, cortical bone cells from Ctsk‐Cre;Trp53^f/f^/Rb1^f/f^ and Ctsk‐Cre mice were first fixed with 1% formaldehyde. Then, sonicated nuclear lysates were isolated and immunoprecipitated with primary anti‐YAP antibody or control rabbit IgG. Precipitated DNA fragments were amplified by qPCR using specific primers to the *Glut1* promoter region (from –1094 to –1100 bp). The primers for ChIP‐qPCR are listed in Table S1.

### 4.12 Calcein labeling and histology

Calcein (20 mg/kg) was injected on Day 2 and Day 5 before 2‐month‐old Ctsk‐Cre, Ctsk‐Cre;Trp53^f/f^, Ctsk‐Cre;Rb1^f/f^, and Ctsk‐Cre;Trp53^f/f^/Rb1^f/f^ mice were sacrificed. After sacrifice, the tibias were collected, fixed in 4% PFA overnight at 4°C, infiltrated with 10% potassium hydroxide (KOH) for 3 days, dehydrated in ethanol and xylene, and then embedded in paraffin. The paraffin sections were prepared at 6 μm thickness and observed under a fluorescence microscope. The mineral apposition rate (MAR) and bone formation rate per bone surface (BFR) were analyzed with a Leica microanalysis system, as we previously reported.^54^

For histological analysis, briefly, femurs were harvested, fixed in 4% PFA overnight at 4°C, decalcified with 14% EDTA in PBS (pH 7.4) for 1 month, and then embedded in paraffin. Six‐micrometer sections of these femurs were prepared, and then H&E and TRAP staining was conducted as we previously reported.^1^, ^56^, ^58^

### 4.13 Bioinformatic analysis

Publicly available human osteosarcoma data from BioProject database (ID: PRJNA539828)^2^ of National Center for Biotechnology Information (NCBI) were used to determine glucose‐related energy metabolism and Glut1 expression. For each gene, significant differences in expression were identified using the two‐tailed, Student's *t*‐test, with *p*‐values <0.05 considered statistically significant. All data were downloaded and analyzed by R packages DESeq2 and ClusterPfofiler.

### 4.14 Statistical analysis

In this study, the analyses of experimental data were conducted and analyzed by the software SPSS 21, and data were reported as mean ± SEM by Student's *t*‐test. The statistical significance of group differences was determined by two‐way ANOVA. The *p*‐values <0.05 were considered significant.

## CONFLICT OF INTEREST

The authors declare that there is no conflict of interest.

## ETHICS STATEMENT

All mice experiments (protocol # 806004) were carried out with the guidelines of the Institutional Animal Care & Use Committee at the University of Pennsylvania.

## AUTHOR CONTRIBUTIONS

Y.Y.S and Y.L. (Yang Li) conceived this study and designed the experiments. Y.L. (Yang Li) and S.T.Y carried out experiments and analyzed data. Y.L. (Yang Liu) analyzed the human osteosarcoma data from BioProject database (ID: PRJNA539828). Y.L. (Yang Li) and Y.Y.S wrote, reviewed and edited the manuscript. Y.Y.S supervised the project.

## Supporting information

Supporting Information

## Data Availability

All the data are available from the corresponding authors upon reasonable request.
