# Supplementary material for: Deletion of Trp53 and Rb1 in Ctsk‐expressing cells drives osteosarcoma progression by activating glucose metabolism and YAP signaling
Source: MedComm (2020). 2022 Apr 22;3(2):e131. doi: 10.1002/mco2.131 (PMC9026232; doi:10.1002/mco2.131)
Supplement: Supplementary file 1 — Supporting Information [file MCO2-3-e131-s001.docx]

**Deletion of Trp53 and Rb1 in Ctsk-expressing cells drives osteosarcoma progression by activating glucose metabolism and YAP signaling**

Yang Li^1^, Shuting Yang^1^, Yang Liu^2^, Shuying Yang^1,3,4^*

^1^Department of Basic & Translational Sciences, School of Dental Medicine, University of Pennsylvania, Philadelphia, PA 19104, USA

^2^College of Fisheries and Life Science, Dalian Ocean University, Dalian 116023, China

^3^Center for Innovation & Precision Dentistry, School of Dental Medicine, School of Engineering and Applied Sciences, University of Pennsylvania, Philadelphia, PA 19104, USA

^4^The Penn Center for Musculoskeletal Disorders, School of Medicine, University of Pennsylvania, Philadelphia, PA 19104, USA

*Correspondence: Dr. Shuying Yang (shuyingy@upenn.edu)

**Running title:** Trp53 and Rb1 prohibit osteosarcoma progression

**Figure Legends**

**Figure S1** Single deletion of Trp53 or Rb1 in Ctsk-expressing cells doesn’t cause osteosarcoma. (A) mRNA expression level in Ctsk^+^ cells as shown. (B) mRNA expression level in osteoclasts as shown. (C) Representative X-ray images of Ctsk-Cre;Trp53^f/f^, Ctsk-Cre;Rb1^f/f^ mice and controls (Ctsk-Cre) at different timepoints as indicated. Error bars were the means ± SEM from three independent experiments. ***P* < 0.01.

**Figure S2** mRNA expression level. (A and B) qRT-PCR analysis of expression of *Trp53* and *Rb1* as indicated after transfection for 48 hr with Ad-GFP or Ad-Cre in the cortical bone cells from Ctsk-Cre; Trp53^f/f^ or/and Rb1^f/f^ mice as indicated. (C) *TEAD1* mRNA level after transfection of scramble siRNA (Control) or TEAD siRNA for 48 hr as indicated. (D and E) *YAP* mRNA level was identified after overexpression or knockdown of YAP for 48 hr in cortical bone cells from Ctsk-Cre;Trp53^f/f^/Rb1^f/f^ mice. Error bars were the means ± SEM from three independent experiments. ***P* < 0.01, ****P* < 0.001.

**Figure S3** mRNA expression level. qRT-PCR analysis of expression of *CYR61* and *CTGF* as indicated. Error bars were the means ± SEM from three independent experiments. ***P* < 0.01.

**Figure S4** Loss of YAP inhibits the osteosarcoma cell growth. The cortical bone cells from Ctsk-Cre;Trp53^f/f^/Rb1^f/f^ and Ctsk-Cre;Trp53^f/f^/Rb1^f/f^/YAP^f/f^ mice were collected and seeded in 96-well plate. After culture as indicated time, the cell proliferation was determined by WST-1 Cell Proliferation Assay Kit (Cayman Chemical, USA). Error bars were the means ± SEM from three independent experiments. ***P* < 0.01.

**Figure S1**


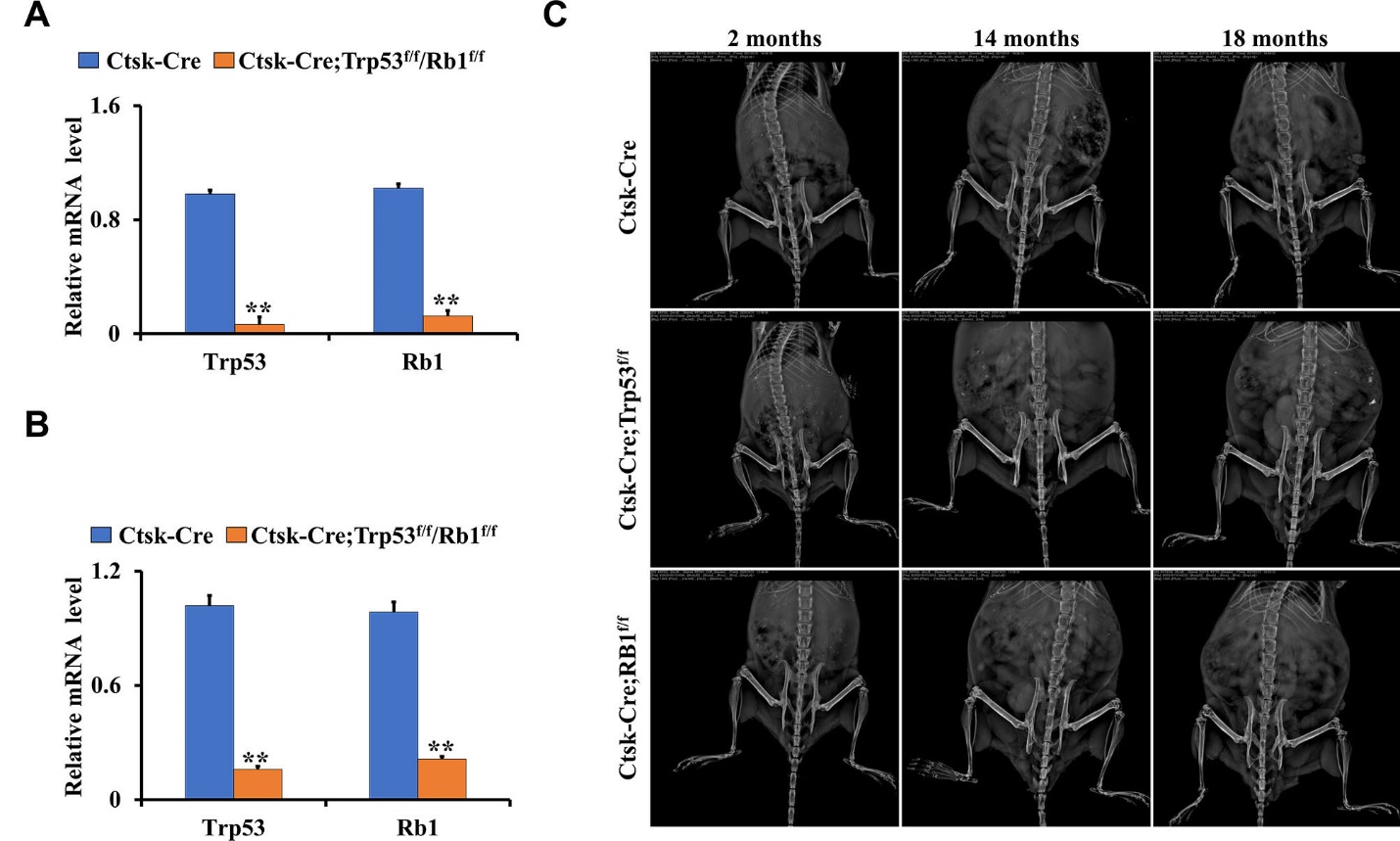


**Figure S2**


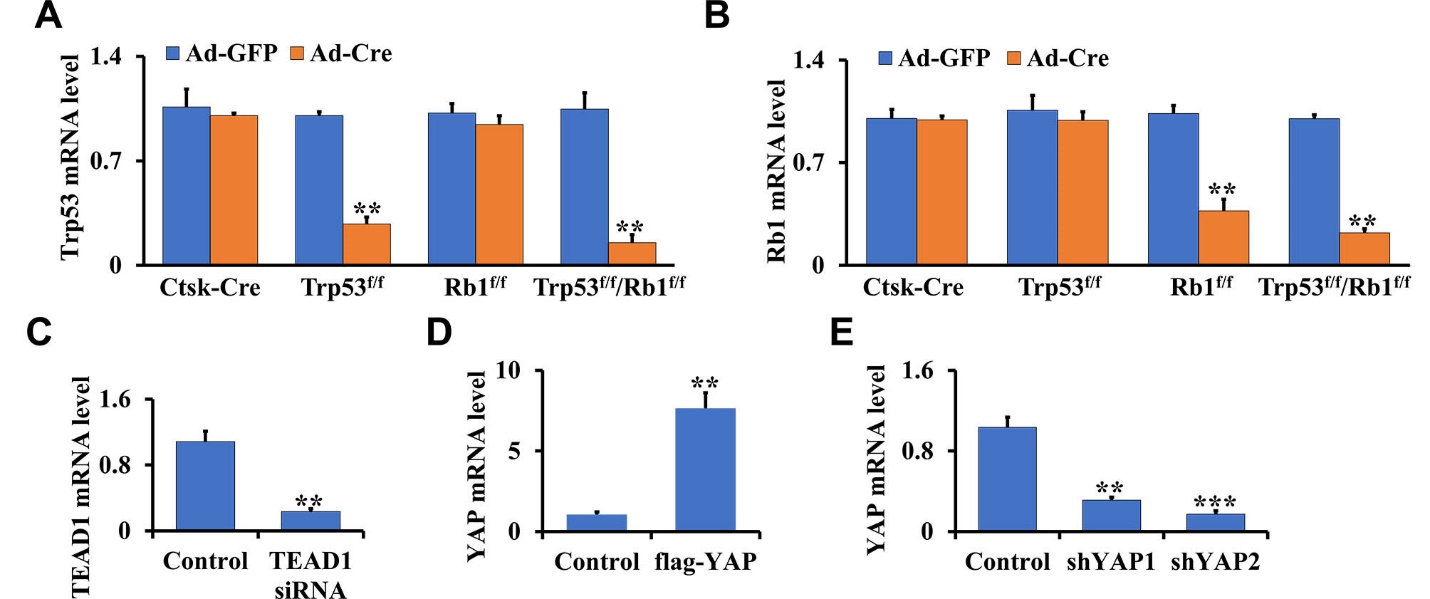


**Figure S3**


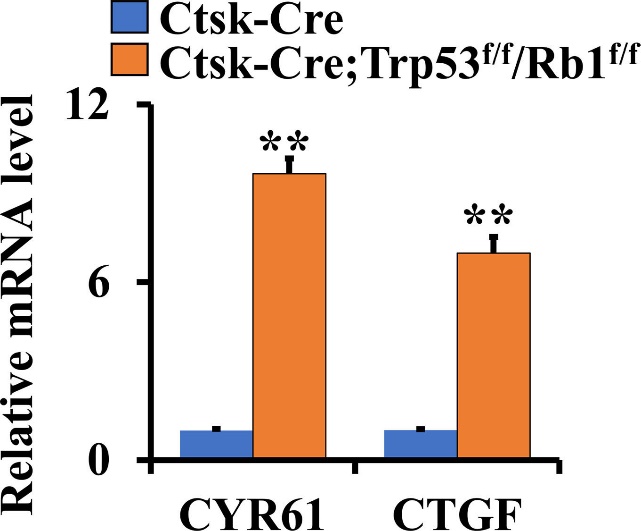


**Figure S4**


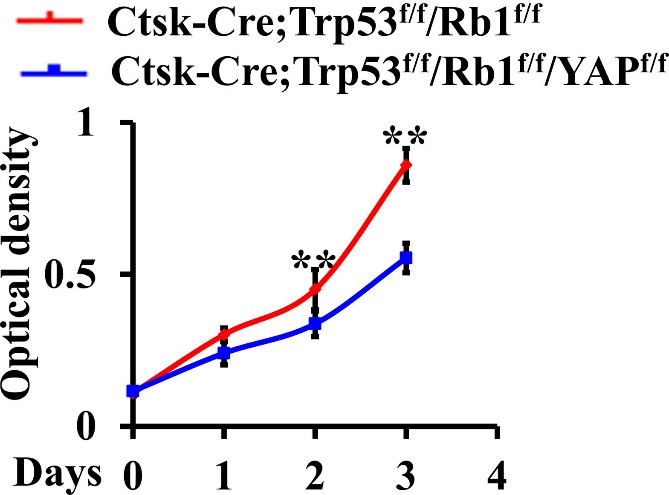


**Supplementary information, Table S1**

| **Gene** | **sequence （5'-3'）** | **Gene** | **sequence （5'-3'）** |
| --- | --- | --- | --- |
| GAPDH-F | CCTGGTCACCAGGGCTGCCATTT | Runx2 | GCCGGGAATGATGAGAACTA |
| GAPDH-R | CGTTGAATTTGCCGTGAGTGGAG | Runx2 | GGACCGTCCACTGTCACTTT |
| YAP-F | GGGGACTCGGAGACCGACTTGGA | ALP-F | AAGGCTTCTTCTTGCTGGTG |
| YAP-R | AGGAGTCGGGCAGCTTGCGAAGC | ALP-R | GCCTTACCCTCATGATGTCC |
| TEAD1-F | CCAGATACATCAAACTCAGGACGG | OSX-F | GGAGGCACAAAGAAGCCATACGC |
| TEAD1-R | GGCGGCTTGAATTTCTCGAACT | OSX-R | TGCAGGAGAGAGGAGTCCATTG |
| Trp53-F | ACTATGGCTTCCACCTGGGC | OCN-F | CTTGGTGCACACCTAGCAGA |
| Trp53-R | GCTGGCAGAATAGCTTATTGAGGG | OCN-R | ACCTTATTGCCCTCCTGCTT |
| Rb1-F | TGGGAGAAAGTTTCATCCGTGG | Glut1-F | GGGCATGTGCTTCCAGTATGT |
| Rb1-R | GCATCTCATCTAGATCAACTGCTGCG | Glut1-R | ACGAGGAGCACCGTGAAGAT |
| HK2-F | TGATCGCCTGCTTATTCACGG | Pfkfb3-F | CTCCCAGCCCGGGGTAAGACTTACA |
| HK2-R | AACCGCCTAGAAATCTCCAGA | Pfkfb3-R | GCTTCACAGCCTCACGCCGATA |
| Pfkfb4-F | CCGACACTCATTGTCATGGTGG | Ldha-F | TGTCTCCAGCAAAGACTACTGT |
| Pfkfb4-R | CACGCCAATCCAGTTGAGGTAC | Ldha-R | GACTGTACTTGACAATGTTGGGA |
| Chip-F | GCCTTGTTCCAGCCCTGCATCAT | CYR61-F | TGTCGCCGTCACCCTTCTCCACTT |
| Chip-R | CCGAGTTTCTTCTGCTGACTGCA | CYR61-R | TTAGCGCAGACCTTACAGCAGCCG |
| CTGF-F | TGCTATGGGCCAGGACTGCA | CTGF-R | AGTTCTCCCAGCTGCTTGGC |
